# Application of aza-BODIPY as a Nitroaromatic Sensor

**DOI:** 10.1021/acsomega.3c02349

**Published:** 2023-07-04

**Authors:** Bleda Can Sadikogullari, Ilayda Koramaz, Berkay Sütay, Bunyamin Karagoz, Ayşe Daut Özdemir

**Affiliations:** Department of Chemistry, Istanbul Technical University, Maslak, 34469 Istanbul, Turkey

## Abstract

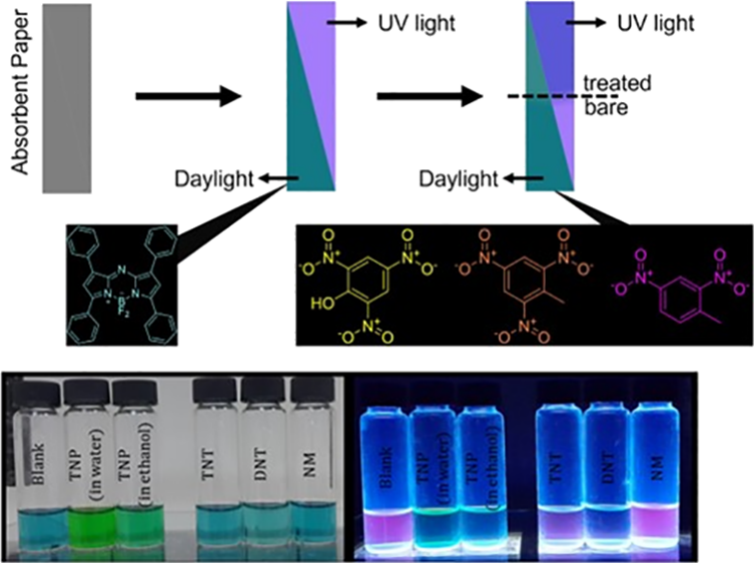

Nitroaromatic explosive detection with high sensitivity and selectivity is requisite for civilian and military safety and the ecosystem. In this study, aza boron dipyrromethene (aza-BODIPY) dye was selected as a fluorescent-based chemosensor against nitroaromatic compounds (NACs) including 2,4,6-trinitrophenol (picric acid, TNP), 2,4,6-trinitrotoluene (TNT), and 2,4-dinitrotoluene (DNT). This dye molecule exhibits sharp fluorescent behavior with high quantum yields beyond the near-infrared region (NIR) and is considered as a potential candidate for the detection of NACs. O’Shea’s approach was used to synthesize tetraphenyl-conjugated aza-BODIPY molecules. Quenching of fluorescence emission of aza-BODIPY at 668 nm after the exposure to NACs was investigated under acetonitrile–water and acetonitrile–ethanol solvent conditions. The quenching responses and its mechanism were examined by considering the Stern–Volmer relationship Stern–Volmer constants (*K*_sv_) for TNP (in water), TNP (in ethanol), TNT, and DNT, which are predicted to be 1420, 1215, 1364, and 968 M^–1^, respectively, all of which are sufficiently above the limit of detection (LOD) values. Thus, the present study opens up the possibility of the usage of aza-BODIPY molecules as a low-cost, light-weight sensor for the detection of NAC explosives.

## Introduction

Detection of the nitroaromatic explosives with high sensitivity and selectivity has vital importance for civilian and military safety and for the ecosystem as well. Explosives are reactive substances with a high potential energy due to the presence of oxidizing and reducing groups in their structures. The most common explosives are nitro-, azide-, peroxo-, and hydrazino-substituted organic compounds.^1^ The variety of industrial applications of such compounds from rocket fuels to dye industries is also responsible for a lot of NO_2_ pollutants which are emitted by the soil and water.^2^ Under the recent developments in science and technology, access to explosives becomes easier, which not only threatens home-land security but also causes an environmental crisis and puts the public health under risk. Many explosives are also known to be biologically active and hazardous to human health. Most of them cause skin and eye irritation, liver damage, and anemia. Especially, exposure to picric acid (TNP) can cause nausea, cyanosis, and cancer, whereas 2,4,6-trinitrotoluene (TNT) causes headache and weakness.^2,3^ The acceptable level of TNT concentration in drinking water is 2 ppb according to the U.S. Environmental Protection Agency (EPA) and the permissible TNP concentration is 2 μM.^3^ As a result, monitoring and detection of NACs, as one of the common explosives, is highly important. For such reasons, a variety of methods have been employed, such as mass spectroscopy (MS), gas chromatography–mass spectroscopy (GC–MS), liquid chromatography–mass spectroscopy (LC–MS), X-ray imaging, and ultraviolet Raman spectroscopy.^4−10^ However, all these methods are of high cost and not suitable in field use. Thus, the techniques for their real-time, on-site detection and their quantification are of great interest, in terms of higher sensitivity and selectivity, in a simpler, cheaper, and faster way. At this point, fluorescent chemosensors appear as a reliable method that can satisfy these needs. Due to their electron-poor nature, explosives can interact with electron-rich fluorescent probes, providing significant fluorescence quenching with electron and charge transfer mechanisms.^11,12^

Small fluorophore molecules^12−14^ as well as fluorophore-tethered homo/copolymers^15−18^ metal–organic frameworks,^19−21^ and nanoparticles^18^ have been widely reported in the literature. Most of these studies utilize pyrene,^16,17,22^ coumarin,^15^ rhodamine,^23^ triphenylamine,^24^ porphyrin,^25^ etc.

On the other hand, the use of aza-BODIPYs (4,4′-difluoro-4-bora-3a,4a,8-triaza-s-indacenes), which can compete with the previously known fluorophore compounds in many aspects including high absorption coefficients, high chemical and photo stabilities, and sharp fluorescence peaks with high quantum yields beyond the near-infrared region^26^ seem to behave as a potential candidate for the detection of NACs. Especially in terms of environmental health, as a near-infrared (NIR)-emitting dye, aza-BODIPY can utilize to monitor accumulation in living bodies. Its ability to be used in monitoring living organisms while having minimal impact on their health is particularly significant and makes it a valuable tool in scientific research and environmental monitoring.^27^ Moreover, the structural modifications of aza-BODIPY molecules are easy to be carried out. In this work, bare aza-BODIPY was synthesized with 4-step O’Shea’s method to investigate its potential for the detection of NACs. The product was treated with several NACs and their fluorescence quenching was examined. As a result, a promising quenching of fluorescence emission of aza-BODIPY after exposure to NACs presence was observed.

## Experimental Section

### Materials

Chemicals used in synthesis are acetophenone (provided from Carlo Erba Reagents, ≥99.0%), ammonium acetate (provided from Merck, ≥98.0%), benzaldehyde (provided from Merck, ≥99.0%), boron trifluoride diethyl ether complex (provided from Fluka, contains 1:1 complex, with Assay 48–52% (BF_3_) GC ≥98.0%), diethylamine (provided from JT Baker, ≥99.0%), hydrochloric acid (provided from Carlo Erba Reagents, ≥37.0% (w/w)), nitromethane (provided from Merck, ≥98.0%), sodium hydroxide (provided from Sigma Aldrich, ≥98.0%), triethylamine (provided from Merck, ≥99.0%), and fluorescence titration 2,4,6-trinitrotoluene ((TNT) provided from Merck, ≥99.0%), 2,4-dinitrotoluene ((DNT), provided from Merck, ≥97.0%), (2,4,6-trinitrophenol ((TNP), provided from Merck, moistened with water, ≥98.0%), nitromethane ((NM), provided from Merck ≥97.0%), and nitroethane ((NE), provided from Merck ≥97.0%) were directly used without purification. Solvents used in synthesis, purification, and fluorescence titration (1-butyl alcohol (provided from Carlo Erba), acetonitrile (provided from Supelco), dichloroethane (provided from Labkim), dichloromethane (technical grade), diethyl ether (provided from isolab), ethyl alcohol (technical grade), hexane (technical grade), and methyl alcohol (technical grade)) were distilled and preserved with molecular sieve 4A (provided from Carl Roth) before use. Deionized distilled water was used in all experiments if needed and all other chemicals were used as received.

### Instrumentation

All NMR spectra were recorded on a Varian spectrometer (500 MHz for ^1^H spectra and 125 MHz, for ^13^C spectra). Proton and carbon chemical shifts are reported in parts per million downfield from tetramethyl silane, TMS. Mass spectra were recorded on Thermo LCQ-Deca ion trap mass instruments (HR-MS). As for optical measurements, UV–vis measurements were taken in a T80 + UV/vis spectrophotometer with quartz cuvettes in the 200–2500 nm range (light path: 10 mm), and fluorescence measurements were carried out utilizing a quartz cell with 10 mm path length via an Agilent Cary Eclipse fluorescence spectrophotometer device at room temperature. During the fluorescence measurements, the excitation wavelength was set as 640 nm; meanwhile, the slit width was adjusted to constant at 5 nm (excitation)/10 nm (emission), and the device voltage was adjusted to 600 V. Finally, the Horiba Jobin Yvon SPEX Fluorolog 3-2iHR (France) instrument was used for recording time-resolved fluorescence measurements in which the source of excitation was NanoLED (France) which excited samples at 670 nm.

### Synthesis of Tetraphenyl-Conjugated aza-BODIPY

**Figure 1:**
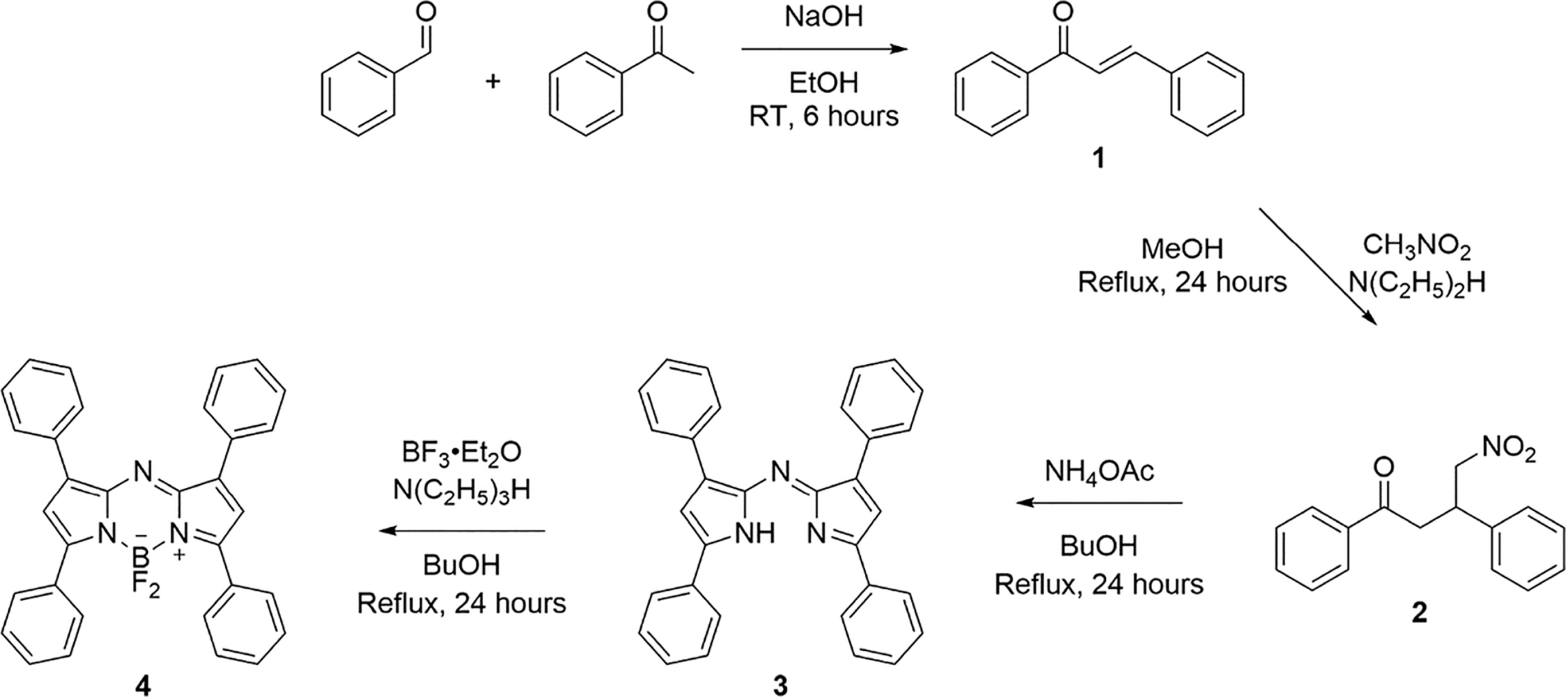
Schematic representation of aza-BODIPY synthesis.

The target sensor was synthesized via O’Shea’s method shown in Figure 1, in which, first, aldol condensation was utilized to obtain *E-*chalcone (**1**) from benzaldehyde and acetophenone. The obtained product was then treated with nitromethane, and the Michael addition (**2**) product was condensed with ammonium acetate to form aza-dipyrromethene (**3**). The resulting tetraphenyl-conjugated aza-dipyrromethene was complexed with boron trifluoride etherate in the basic medium to yield target tetraphenyl conjugated aza-BODIPY (**4**).^26,28,30^

#### *E*-Chalcone **(1)**

Benzaldehyde (5.00 mL, 0.05 mol) in EtOH (30 mL) was added directly to the solution of NaOH (4.90 g, 0.12 mol) in water (50 mL). To that mixture, acetophenone (5.72 mL, 0.05 mol) in ethyl alcohol (10 mL) was added via a dropping funnel. The reaction mixture was stirred at room temperature for 6 h. The mixture was continued to be stirred for additional 1 h at 0 °C to precipitate the product. Precipitated *E-*chalcone was filtered and washed with ice-cold ethyl alcohol and obtained as pale-yellow powder. For further purification, recrystallization from alcohol was done (Yield: 97.80%). ^1^H NMR (500 MHz, CDCl_3_) δ: 8.04 (dd, *J = 7.4, 1.7* Hz, 2H), 7.83 (d, *J = 15.7* Hz, 1H), 7.66 (dd, *J = 6.6, 3.0* Hz, 2H), 7.61 (t, *J = 5.00* Hz, 1H), 7.58–7.49 (m, 3H), 7.43 (dd, *J = 5.0, 1.9* Hz, 3H). ^13^C NMR (125 MHz, CDCl_3_) δ: 190.56, 144.85, 138.24, 134.91, 132.80, 130.57, 129.16, 128.65, 128.53, 128.47, 122.12. (Supplementary Material Figures S1 and S2).

#### 4-Nitro-1,3-diphenylbutan-1-one (**2**)

*E-*Chalcone (2.5 g, 0.012 mol) was dissolved in hot methyl alcohol (30 mL). To that solution, dimethylamine (3.21 mL, 0.06 mol) and nitromethane (6.21 mL, 0.06 mol) were added, and the mixture was refluxed for 24 h. After completion, the reaction was allowed to cool down to room temperature and poured into 1 N HCl solution. The product was precipitated out as off-white, gray powder, and filtered. The cure product was washed with ice-cold methyl alcohol. For further purification, recrystallization from alcohol was done. (Yield: 92.07%). ^1^H NMR (500 MHz, CDCl_3_) δ: 7.93 (dd*, J = 5.0, 1.7* Hz, 2H), 7.58 (t, *J = 5.0* Hz, 1H), 7.47 (t, *J = 5.0* Hz, 2H), 7.35 (t, *J = 5.0* Hz, 2H), 7.32–7.27 (m, 3H), 4.85 (dd, *J = 12.5, 6.6* Hz, 1H), 4.71 (dd, *J = 12.5, 8.0* Hz, 1H), 4.25 (q, *J = 10* Hz, 1H), 3.54–3.40 (m, 2H). ^13^C NMR (125 MHz, CDCl_3_) δ: 196.83, 139.12, 136.38, 133.57, 129.08, 128.75, 128.02, 127.89, 127.46, 79.57, 41.53, 39.29. (Supplementary Material Figures S3 and S4).

#### (*Z*)-*N*-(3,5-Diphenyl-1*H*-pyrrol-2-yl)-3,5-diphenyl-2*H*-pyrrol-2-imine (**3**)

Solutions of 4-nitro-1,3-diphenylbutan-1-one (**2**) (1.00 g, 3.71 mmol) and ammonium acetate (9.99 g, 0.13 mol) in n-butyl alcohol (25 mL) were saturated with nitrogen and refluxed for 24 h under nitrogen. The target product was precipitated out during the course of the reaction. The reaction was cooled to room temperature, filtered, and the isolated solid was washed with cold ethanol to yield the product as a blue-black solid. The product was immediately used for the next procedure without any extra purification. (Yield: 44.04%). ^1^H NMR (500 MHz, CDCl_3_) δ: 8.08 (dd, *J = 6.6, 1.3* Hz, 4H), 7.97 (dd, *J = 6.6, 1.3* Hz, 4H), 7.56 (t, *J = 5.0* Hz, 4H), 7.52–7.41 (m, 6H), 7.38 (t, *J = 5.0* Hz, 4H), 7.22 (s, 2H), NH cannot be observed. ^13^C NMR (125 MHz, CDCl3) δ: 155.11, 149.60, 142.66, 133.71, 132.19, 130.08, 129.15, 129.08, 128.26, 128.01, 126.56, 114.92. (Supplementary Material Figures S5 and S6).

#### aza-BODIPY (**4**)

The solution of the aza-dipyrromethene ligand (3) (1.5 g, 3.34 mmol) in dry dichloroethane (80 mL) was prepared, followed by the addition of triethylamine (0.24 mL, 1.40 mmol). The resulting solution was stirred under a nitrogen atmosphere until the reflux temperature was reached. Subsequently, boron trifluoride diethyl etherate (0.31 mL, 2.51 mmol) was added, and the reaction mixture was refluxed for 1 day under nitrogen. Upon completion, the mixture was washed with water (2 × 80 mL), dried over magnesium sulfate, and evaporated to dryness. Purification of the product was achieved by column chromatography on silica gel, eluting with a mixture of dichloromethane and hexane (3:1). The obtained product was further subjected to rapid evaporation of diethyl ether for crystallization (Yield: 66%). ^1^H NMR (500 MHz, CDCl_3_) δ: 8.11–8.03 (m, 8H), 7.55–7.41 (m, 12H), 7.06 (s, 2H). ^13^C NMR (125 MHz, CDCl_3_) δ: 159.57, 145.61, 144.22, 132.31, 131.60, 130.93, 129.64, 129.40, 128.66, 128.62, 119.17, 119.15, 119.12. ^19^F NMR (471 MHz, CDCl_3_) δ: −131.40 (q). HR – MS (*m*/*z*): [M + H]^+^ (C_32_H_23_N_3_BF_2_) theoretical: 498.19476; measured: 498.19669. (Supplementary Material Figures S7–S10).

### Fluorescence Detection of NACs

Fluorescence detection capabilities of each NAC were examined individually at room temperature. Titration studies are then carried out with an aliphatic nitro compound which is nitromethane (NM) and nitroethane (NE). For that reason, 1 mM solution of aza-BODIPY in acetonitrile was prepared and UV–Vis measurements were completed. As a result, the maximum absorption wavelength was captured at 642 nm, which is then used as the excitation wavelength in the fluorescence analysis (Supplementary Material, Figure S11). During fluorescence measurements, the ideal concentration was determined as 2.5 × 10^–6^ M according to the fluorescence calibration (Supplementary Material, Figure S12). Solutions (1 mM) of each nitro aliphatic/aromatic were prepared in EtOH (and for TNP in water as well), and titration studies were carried out by direct addition of these stocks.

## Results and Discussion

### Choice of the Material

As a known fluorophore, aza-BODIPY dyes are commonly studied in biological applications as sensitizers and sensors^29^ due to their NIR region emission and excitation capabilities.^30,31^ Besides, aza-BODIPYs can be synthesized from simple compounds that are easily available and can be modified by various methods, which makes it very easy to modify according to the application. Nevertheless, other areas of applications are still fairly new for aza-BODIPY dyes.

It is known that the most common interaction for the examples of explosive sensors in the literature is π–π interactions^16,19,32,33^ As an electron-rich moiety, the possibility of getting significant results encouraged us to try against TNP. The first optical analysis with TNP shows promising quenching of fluorescence emission, which motivated us to investigate further nitro-aromatics. As a result, herein, it is presented the very first application of aza-BODIPY as an NAC sensor.

### Detection of Nitro-Aromatics

Fluorescence titration was carried out at least 3 times to investigate the optical detection of nitro compounds via fluorescence quenching of aza-BODIPY. Additionally, each titration was repeated with blank samples to ensure whether the quenching is only caused by dilution or the interaction between the analyte and the sensor. The standard deviation (σ_10_) was calculated from intensities of bare sensor (*I*_0_) in repeated fluorescence measurements, which is then used to calculate LOD (Supplementary Material, Figure S14 and Table S1).

**Figure 2:**
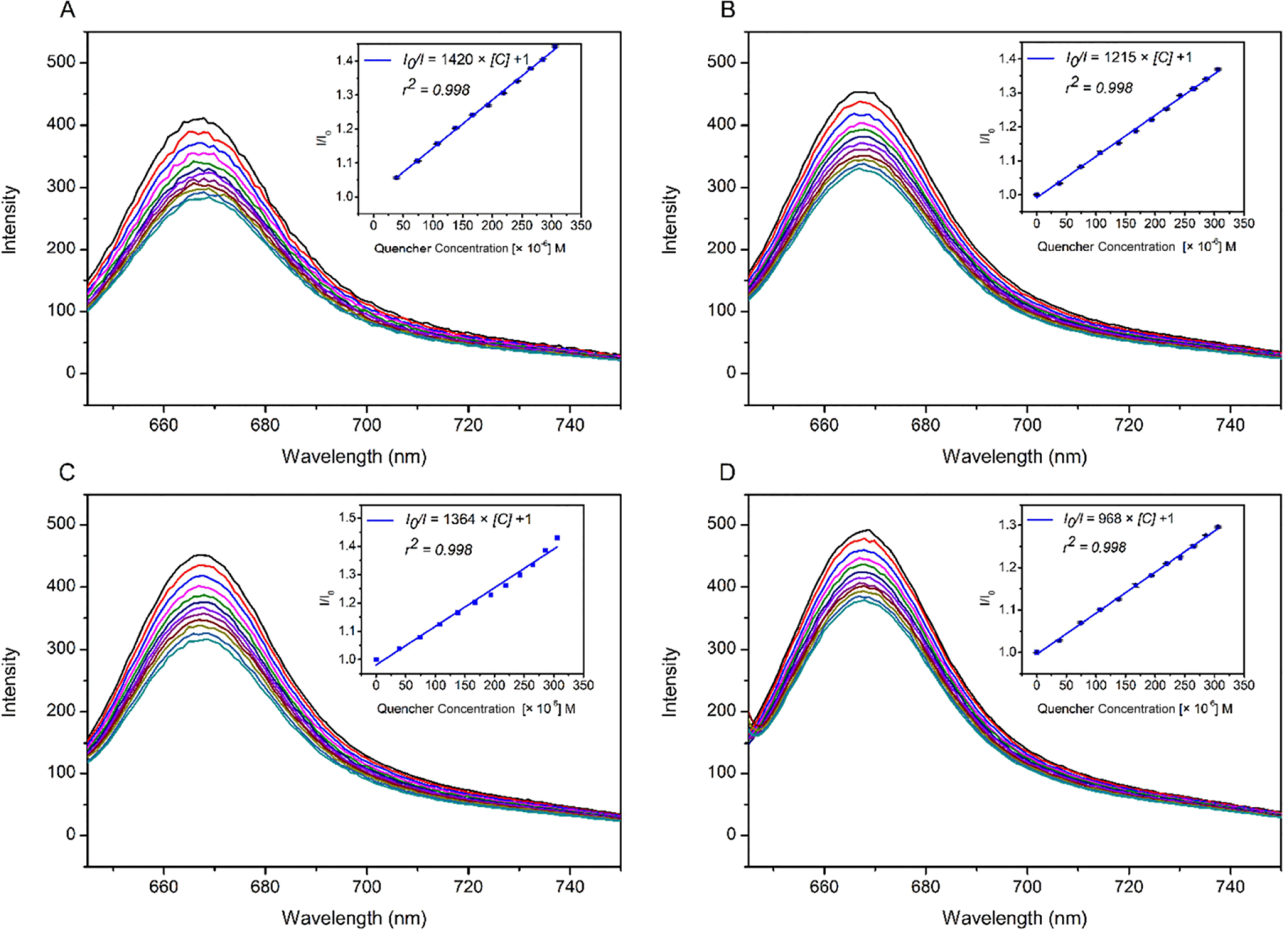
Fluorescence quenching spectra of titrations of aza-BODIPY in acetonitrile with a solution of TNP in water (A), TNP in ethanol (B), TNT in ethanol (C), and DNT in ethanol (D). Stern–Volmer plots (*I*_0_/*I* vs quencher concentration in molarity, the intercept value was set to 1) were also shown.

**Figure 3:**
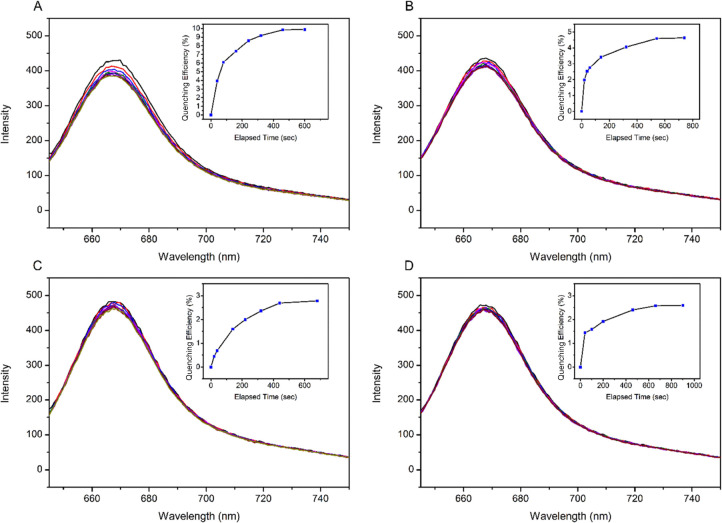
Quenching efficiency of TNP in water (A), TNP in ethanol (B), TNT in ethanol (C), and DNT in ethanol (D).

During the course of titration studies, 2.5 × 10^–6^ M of aza-BODIPY solution in acetonitrile was treated with 50 μL of 1 mM solution of each explosive. Within the scope of the present study, Stern–Volmer graphs are plotted in Figure 2, and the *K*_sv_ values were found as the slope of the regressed graph. Resulting *K*_sv_ values proved the interaction between the aza-BODIPY and the nitroaromatics. The studies were completed without exceeding 200 equivalents of explosive in any titration by considering the fast reaction time of explosive and the aza-BODIPY in given media (Figure 3).

In the time–response graph (Figure 3), a quenching of fluorescence, provided with 16 equimolar explosives, emission was observed with a rapid decline within 2 min. For that amount of quencher, the maximum quenching efficiency was found to be approximately 5% within the first 2 min and no significant increase has been observed in the remaining time. Additionally, the highest quenching efficiency was observed in TNP (in water) compared to the other explosives. Moreover, for overall quenching in fluorophore intensity, no more than 40% decrease was observed in either quencher where TNP (in water) has the greatest value among all.

**Figure 4:**
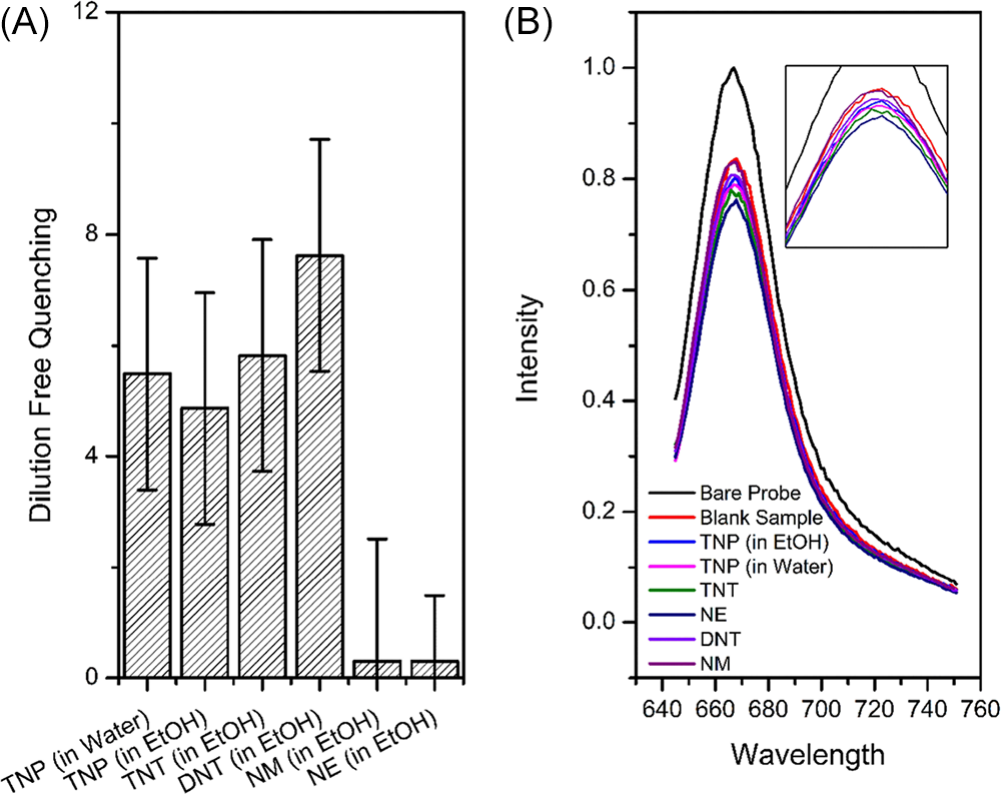
Comparative quenching of NAC free from dilution (A) and corresponding fluorescence spectrum (B).

As a complementary study, aza-BODIPY was treated with nonaromatic NM and NE compounds for further investigation. Considering the fact that with the addition of a quencher with 50 μL increments, the effect of dilution becomes significant in fluorescence quenching. In order to eliminate this effect and observe the interaction with the explosive more clearly, titrations were carried out using NE and NM along with blind titrations against the solvent. As a result, quenching caused by the addition of nonaromatic compounds seems to be caused only from dilution but nothing else, which supports the idea of the interaction of dye and the explosive mostly from π–π interactions (Figure 4). Moreover, to these results, when correction against dilution is done, DNT has a greater impact on quenching (Figure 4). On the other hand, TNP in both media had higher *K*_sv_ values and greater impact on quenching overall (Figure 2). The reason can be addressed with an explanation on the structure of the aza-BODIPY molecule and the effect of media over quenchers. The DNT molecule has a smaller structure compared to other nitro aromatics, so it can interact with the probe more easily. Considering the freely rotating phenyls attached to the probe in the solvent, the importance of this effect increases exponentially in the solvent environment. On the other hand, the more easily soluble structures of TNP and TNT in the environment increased their dispersion in the media and led to higher quenching. As seen from Figure 4, the aza-BODIPY moiety is suitable to act as a nitroaromatic sensor. Considering the easy modifiability of such dyes, the possibility to functionalize according to the target quenchers will provide significant advancements and enhancements in this specific application field.

In an effort to understand all the results obtained, the *K*_sv_ and LOD values are given in Table 1. The best quenching response of aza-BODIPY against TNP (in water) can be explained by the high solubility of TNP in water, which provides easy π–π interactions between the dye molecule and the explosive.^33^

**Table 1:** Summary of Fluorescence Titration Results

explosive (media)	*K*_sv_ (M^*1^)	LOD (×10^–6^ M)	interaction time (s)	time taken for full quenching (min)
TNP (water)	1420	2.32	80	7
TNP (EtOH)	1215	2.32	140	9
TNT (EtOH)	1364	2.14	140	7
DNT (EtOH)	968	2.54	40	11

### Use as a Chemosensor

In light of these findings, studies have been carried out to prove visually the quenching of aza-BODIPY dissolved in acetonitrile with NACs, and surface-impregnated analysis was performed as an attempt to solid-state applications.

**Figure 5:**
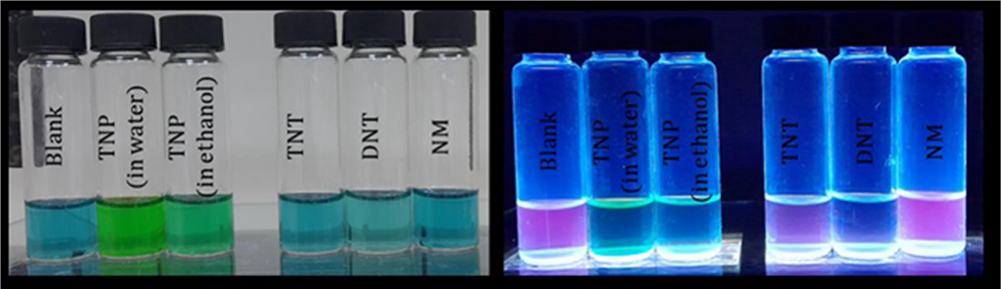
Visualization of quenching upon addition of NACs.

For this purpose, 12 mL glass vials, each containing 2.5 mL, 5 × 10^–3^ M aza-BODIPY in acetonitrile, were treated with 0.5 mL saturated solutions of TNP in ethanol and water, TNT, DNT, and NM in ethanol. For the comparison, 0.5 mL of ethanol was added to the blank vial, and each sample was vortexed for a minute. According to photographs, under UV light (635 nm), TNP causes total quenching, whereas TNT and DNT cause limited quenching over emission. NM caused no significant change after treatment. On the other hand, no significant color change was observed in day light except for TNP, which was caused due to the yellow color of solution (Figure 5).

**Figure 6:**
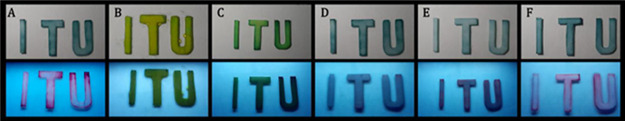
Visual image of surface-impregnated study (daylight sample upper row, UV light lower row, blank sample (A), TNP in water (B), TNP in ethanol (C), TNT in ethanol (D), DNT in ethanol (E), and NM in ethanol (F).

As for an attempt to use as a surface-impregnated application, the letters “ITU” have been cut off using an absorbent paper, after which a crystallization dish is placed filled with 5 × 10^–3^ M aza-BODIPY in acetonitrile. After a night, all solvent was evaporated under a fume hood, and coated letters were treated with the saturated solution of NACs. After treatment, instant quenching was observed except for NM, which is expected due to lack of π–π interactions (Figure 6).

Visual imaging clearly shows that strongly electron-withdrawing groups result in quicker contact; after treatment, instant quenching was observed except for NM, which is expected due to the occurrence of the donor-acceptor π–π interaction. The yellowish color of the TNP sample is due to its natural color, which is easily seen when comparing TNT to DNT samples (Figures 5 and 6).

### Computational Details

**Figure 7:**
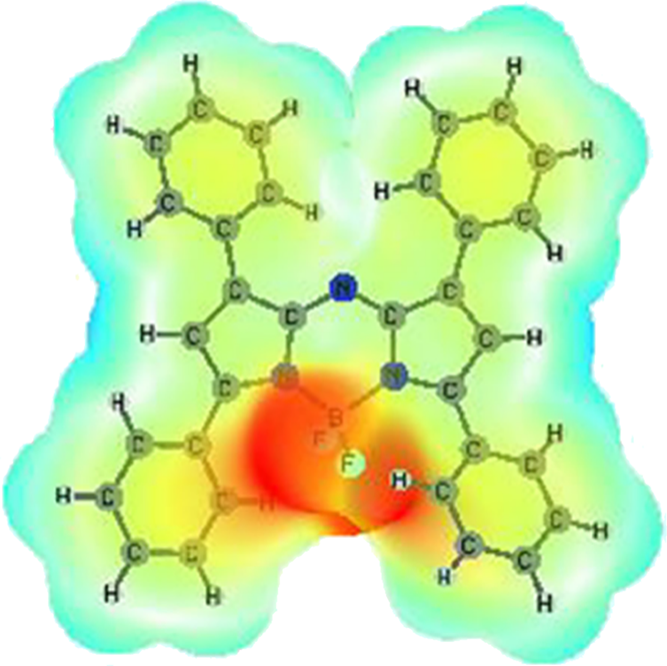
Optimized geometry and molecular ESP map of aza-BODIPY molecules.

The structures of the present molecules were modeled by using density functional theory (DFT). All computations including geometry optimizations were performed at the M06-2X/6-31G(2df, 2pd) level of theory in the Gaussian 16 program package.^34^ The optimized geometry and the related molecular electrostatic potential map (ESP) of aza-BODIPY molecules are shown in Figure 7.

**Figure 8:**
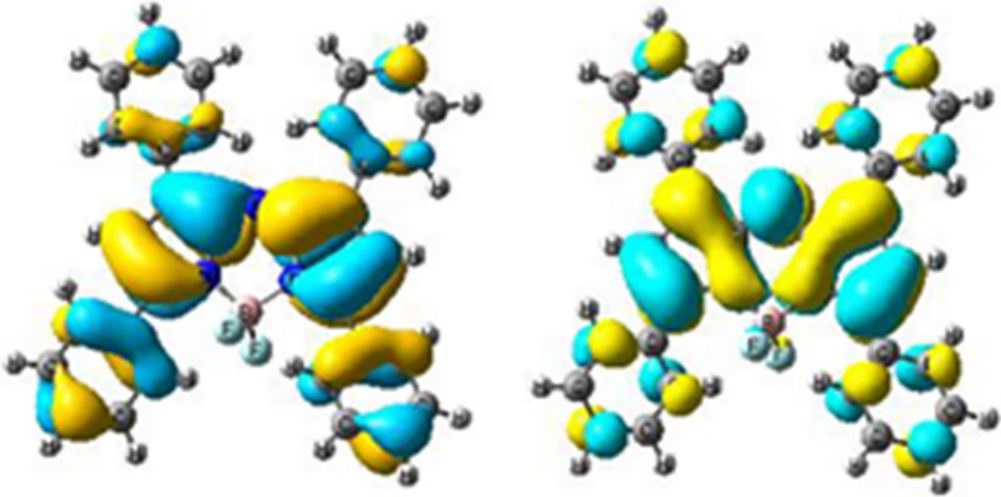
HOMO (left) and LUMO (right) frontier orbitals of the aza-BODIPY molecule.

The frontier molecular orbitals of aza-BODIPY molecules are also shown in Figure 8. The highest occupied molecular orbital (HOMO) orbital was found to be localized on the central unit and partially delocalized over the phenyl groups while the LUMO orbital was mainly located on the central acceptor unit.

**Figure 9:**
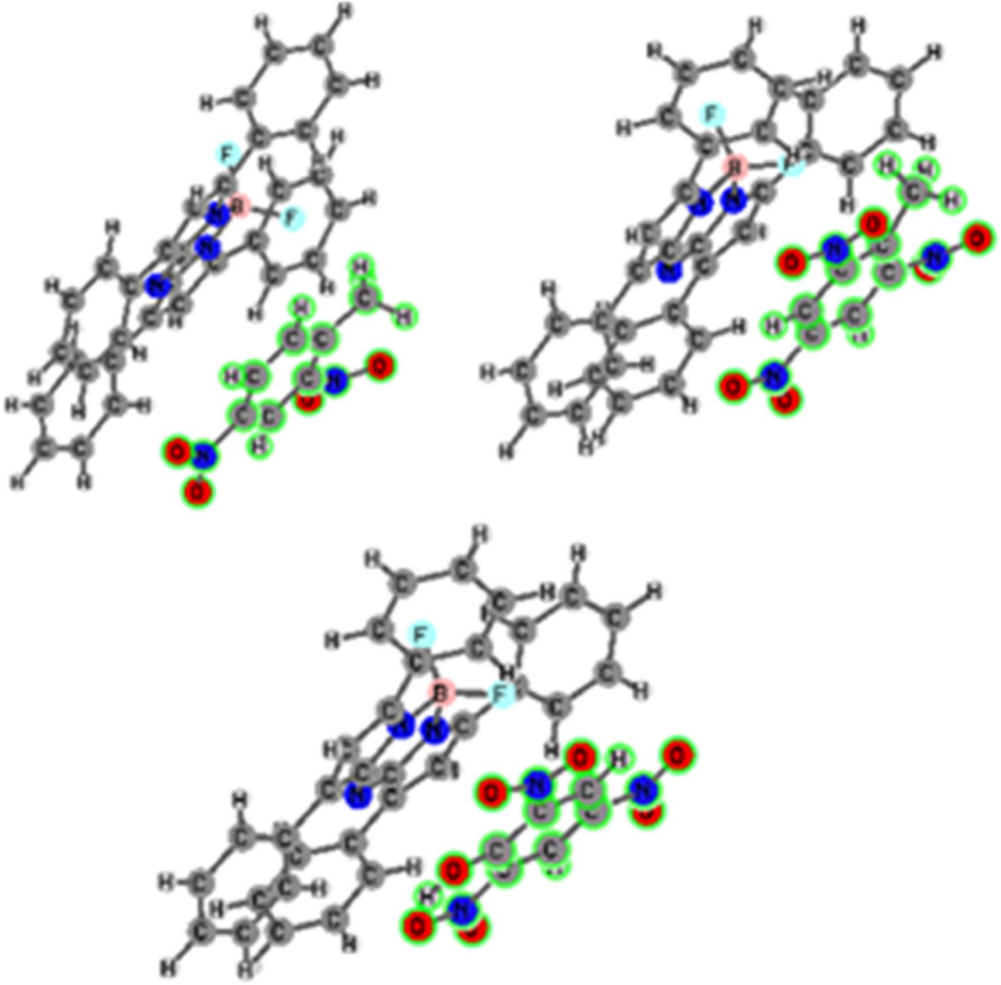
Geometries of the molecular probe-explosive complexes (2,4-DNT complex (top-left), TNT complex (top-right), and TNP complex (bottom)).

The intermolecular interactions of NACs with the aza-BODIPY molecule were studied and the minimum energy configurations of the molecular complexes of aza-BODIPY with NACs are found as shown in Figure 9.

**Figure 10:**
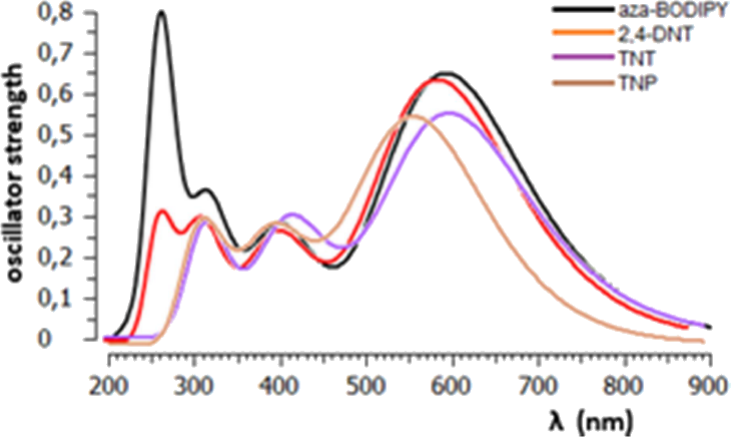
Theoretical emission spectra of aza-BODIPY with or without NACs.

π–π Interactions are found prominent in all complexes. Interaction energies were corrected by the inclusion of the basis set superposition error (BSSE) and were predicted as −12.6, −15.3, and −13.7 kcal mol^–1^ for 2,4-DNT, TNT, and TNP complexes, respectively. TNT and TNP interact with aza-BODIPY molecules stronger than 2,4-DNT. The strongest interaction, which was found in the TNT complex, may be attributed to an additional CH/π type weak noncovalent bonding. The effect of those interactions on the quenching mechanism was further investigated. For that purpose, time-dependent DFT (TD-DFT) calculations were carried out to compute the theoretical emission spectra of the studied systems, Figure 10.

The fluorescence quenching is predicted to be higher in the case of TNP and TNT molecules which make stronger interactions with the probe. Especially, the oscillator strength shows 22% and 15% decrease in the presence of TNP and TNT molecules, respectively. The higher *K*_sv_ value for TNP could be attributed to the higher energy difference between the lowest unoccupied molecular orbital (LUMO) of the probe and TNP compared to other NACs, (Supplementary Material, Table S2). Static quenching may be confirmed by the ground-state molecular complex formation via π–π interactions (Figure 9). The absorption spectra of the probe with or without NACs also exhibit the signal of such a process and thus confirm this result by a shift in the absorption maxima of the probe after to be exposed to NACs (Supplementary Material, Figure S15). On the other hand, there is not a considerable spectral overlap between the emission spectrum of aza-BODIPY with the absorption spectra of NACs, which also supports the static quenching process (Supplementary Material, Figure S16).

## Conclusions

In this study, aza-BODIPY was synthesized successfully and utilized as a chemosensor for NACs for the first time to our knowledge. According to the *K*_sv_ and LOD values, it was found that the aza-BODIPY molecule can be utilized as a low-cost, light-weight sensor for the detection of NACs, especially for the TNP explosive. For the application as a chemosensor, aza-BODIPYs can be easily applied either as a solid surface or in solution for the detection.

Based on the *K*_sv_ and response time graphs, it can be observed that the presence of π delocalization in nitro-bearing compounds leads to the quenching of aza-BODIPY fluorescence. This unique quenching phenomenon presents an opportunity for the detection of such compounds, offering high potential for selectivity specifically against TNP and TNT. The findings of this study highlight the novel and promising application of easily modifiable aza-BODIPY in the field of explosive detection. This research not only introduces a new avenue for detection but also holds promise for further advancements and improvements in this particular application domain.

Among these specific application areas, bioaccumulation stands out. We believe that due to its low toxicity and high lipophilicity, aza-BODIPY holds advantages over other dyes in this regard. Additionally, its easily modifiable structures enable convenient attachment to polymeric chains, facilitating the enhancement of selectivity and effectiveness. Furthermore, the utilization of aza-BODIPY as sensors that offer ease, speed, and reliable results, even eliminating the hassle of sample preparation in aqueous environments, is highly plausible.

## Supporting Information Available

Details of synthesis; characterization; and computational details (PDF)
