# Supplementary material for: Application of aza-BODIPY as a Nitroaromatic Sensor
Source: ACS Omega. 2023 Jul 4;8(28):25254–61. doi: 10.1021/acsomega.3c02349 (PMC10357534; doi:10.1021/acsomega.3c02349)
Supplement: Supplementary file 1 — ao3c02349_si_001.pdf [file ao3c02349_si_001.pdf]

Supporting Information File

"Application of aza-BODIPY as a nitroaromatic sensor"

Bleda Can Sadikogullari, Ilayda Koramaz, Berkay Sütay, Bunyamin Karagoz*, Ayşe Daut Özdemir*

Istanbul Technical University, Faculty of Science and Letters, Department of Chemistry, Maslak, 34469 Istanbul, Turkey
* Corresponding authors: karagozb@itu.edu.tr (Karagöz B.); daut@itu.edu.tr (Daut Özdemir A.)

Table of Contents

Table of Contents 1
List of Figure and Tables 1
1 Materials and instrumentation 1
1.1 Materials 1
1.2 Instrumentation 1
2 Characterization of Synthesized Molecules 2
2.1 Synthesis of the sensor 2
2.2 Optical characterization of the sensor 9
Absorption and Emission Spectra of the sensor. 9
Fluorescence Lifetime Decay 11
2.3 Fluorescence titration optimization 12
Limit of Detection. 12
2.4 Computational Details 13
3 References 15

List of Figure and Tables

Figure S 1. ^{1}H NMR spectrum of *E*-Chalcone. 3
Figure S 2. ^{13}C NMR spectrum of E-Chalcone. 4
Figure S 3. ^{1}H NMR spectrum of 4-nitro-1,3-diphenylbutan-1-one. 4
Figure S 4. ^{13}C NMR spectrum of 4-nitro-1,3-diphenylbutan-1-one. 5
Figure S 5. ^{1}H NMR spectrum of tetraphenyl conjugated azadipyrromethene 5
Figure S 6. 13C NMR spectrum of tetraphenyl conjugated aza-dipyrromethene 6
Figure S 7. ^{1}H NMR spectrum of tetraphenyl conjugated aza-BODIPY. 7
Figure S 8. ^{13}C NMR spectrum of tetraphenyl conjugated aza-BODIPY. 8
Figure S 9. ^{19}F NMR spectrum of tetraphenyl conjugated aza-BODIPY. 9
Figure S 10. HR-MS spectra of aza-BODIPY. 9
Figure S 11. UV-Vis spectrum of aza-BODIPY in acetonitrile (Concentration between 10.00-30.00 μM). 10
Figure S 12. Fluorescence spectrum of aza-BODIPY in acetonitrile ($\lambda_{excitation}$ = 642 nm, concentration between Concentration between 0.25-3.00 μM) 11
Figure S 13. Fluorescence lifetime of aza-BODIPY. 12
Figure S 14. Calibration curves of titration of aza-BODIPY in acetonitrile with a solution of TNP in water (A), TNP in ethanol (B), TNT in ethanol (C) and DNT in ethanol (D). 13
Figure S 15. Theoretical absorption spectra of aza-BODIPY with or without NACs. 14

Figure S 16. Spectral overlap between the theoretical fluorescence spectrum of aza-BODIPY with the absorption spectra of NACs. 15

Table S 1. Limit of Detection Values. 14
Table S 2. The frontier orbital energies for aza-BODIPY and NACs calculated at B3LYP level of theory in polarized double zeta basis (in eV). 15

1 Materials and instrumentation

1.1 Materials

Chemical used in synthesis (acetophenone (provided from Carlo Erba Regeants, ≥99.0%), ammonium acetate (provided from Merck, ≥98.0%), benzaldehyde (provided from Merck, ≥99.0%), boron trifluoride diethyl ether complex (provided from Fluka, contains 1:1 complex, with Assay 48-52 % (BF_3) GC ≥98.0%), diethylamine (provided from JT Baker, ≥99.0%), hydrochloric acid (provided from Carlo Erba Regeants, ≥ 37.0% (w/w), nitromethane (provided from Merck, ≥98.0%), sodium hydroxide (provided from Sigma Aldrich, ≥98.0%), triethylamine (provided from Merck, ≥99.0%)), and fluorescence titration (2,4,6-Trinitrotoluene ((TNT) provided from Merck, ≥99.0%), 2,4-Dinitrotoluene ((DNT), provided from Merck, ≥97.0%), (2,4,6-Trinitrophenol ((TNP) provided from Merck, moistened with water, ≥98.0%), were directly used without purification. Solvents used in synthesis, purification, and fluorescence titration (1-butyl alcohol (provided from Carlo Erba), Acetonitrile (provided from Supelco) dichloroethane (provided from Labkim), dichloromethane (technical grade), diethyl ether (provided from isolab), ethyl alcohol (technical grade), hexane (technical grade), methyl alcohol (technical grade)) were distilled and preserved with molecular sieve 4A (provided from Carl Roth) before use. Deionized distilled water was used in all experiments if needed and all other chemicals were used as received.

1.2 Instrumentation

All NMR spectra were recorded on a Varian spectrometer (500 MHz for ^{1}H spectra and 125 MHz, for ^{13}C spectra). Proton and carbon chemical shifts are reported in parts per million downfield from tetramethyl silane, TMS. Mass spectra were recorded on Thermo LCQ-Deca ion trap mass instruments (HR-MS). As for optical measurements, UV-Vis measurements were taken in a T80 + UV/vis spectrophotometer with quartz cuvettes in the 200-2500 nm range (light path: 10 mm), and fluorescence measurements were carried out utilizing quartz cell with 10 mm path length *via* Agilent Cary Eclipse Fluorescence Spectrophotometer device at room temperature. During the fluorescence measurements, the excitation wavelength was set as 642 nm meanwhile the slit width was adjusted to constant at 5 nm (excitation) / 10 nm (emission) and the device voltage was adjusted to 600V. Lastly, The Horiba Jobin Yvon SPEX Fluorolog 3-2iHR (France) was used for recording time-resolved fluorescence measurements in which the source of excitation was NanoLED (France) which excited samples at 670 nm.

2 Characterization of Synthesized Molecules

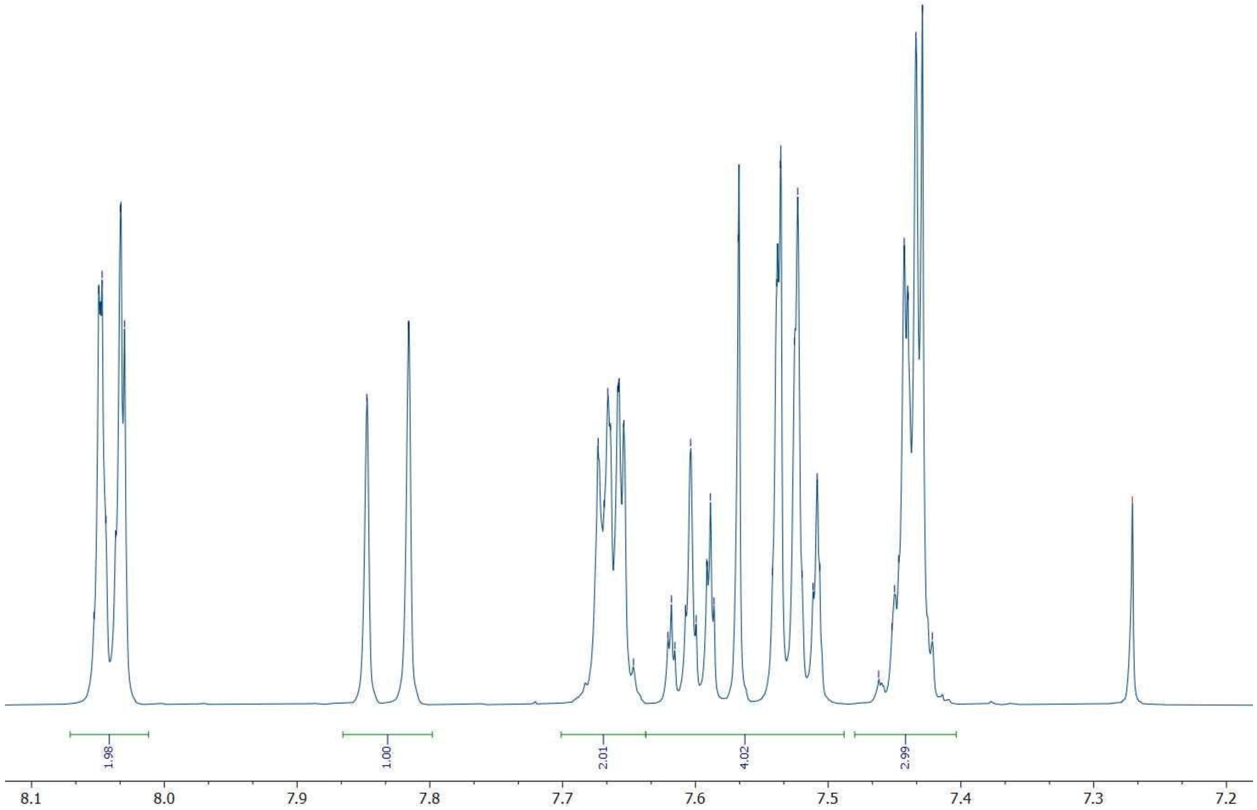

Figure S 1. ^{1}H NMR spectrum of *E*-Chalcone.

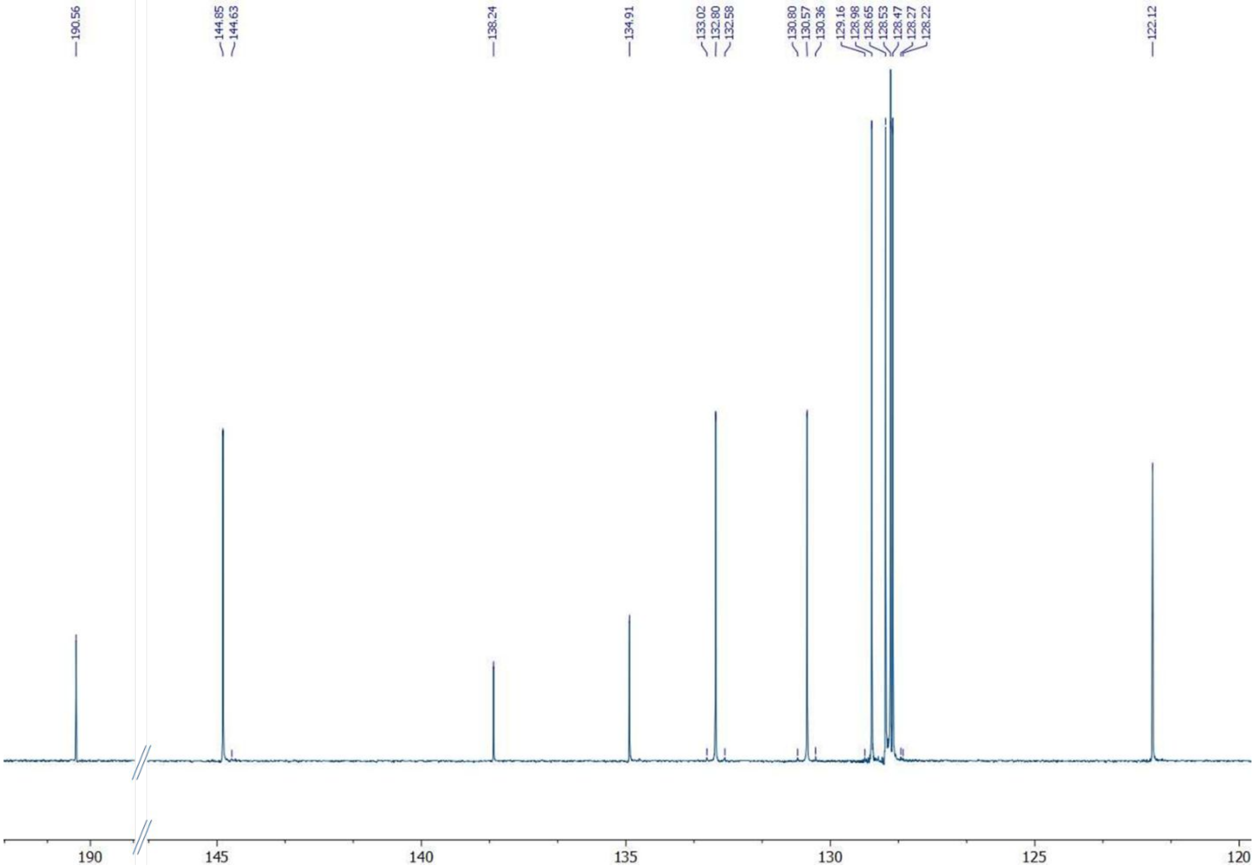

Figure S 2. ^{13}C NMR spectrum of E-Chalcone.

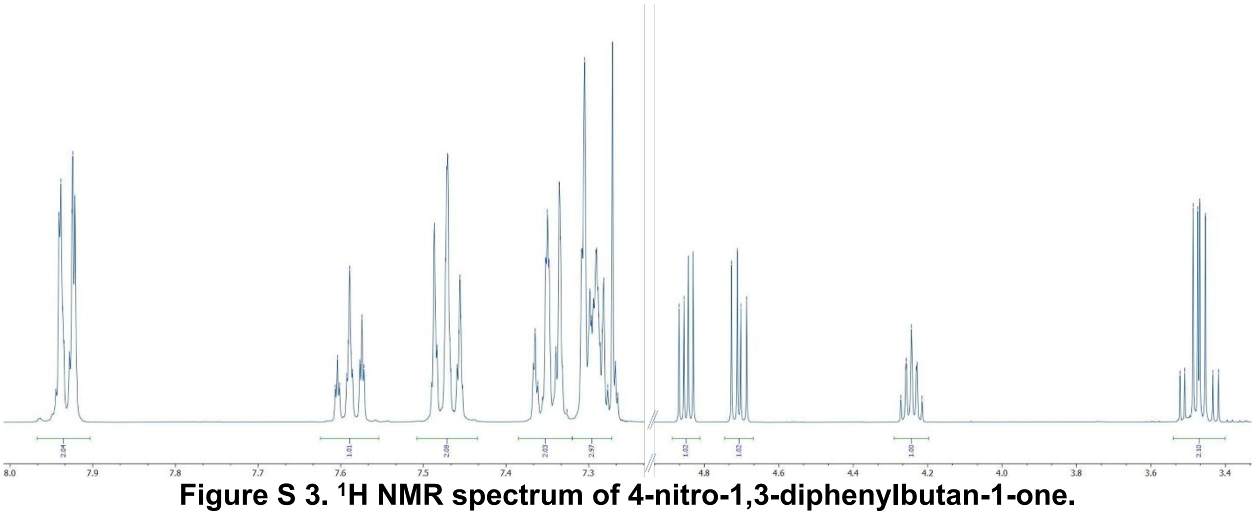

Figure S 3. ^{1}H NMR spectrum of 4-nitro-1,3-diphenylbutan-1-one.

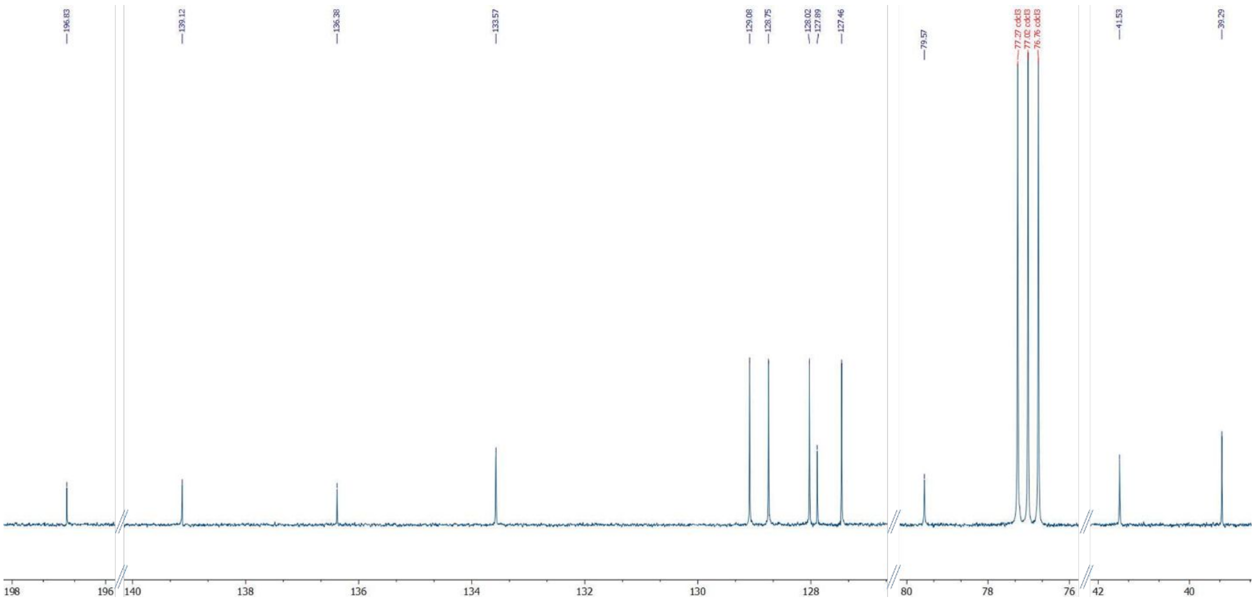

Figure S 4. ^{13}C NMR spectrum of 4-nitro-1,3-diphenylbutan-1-one.

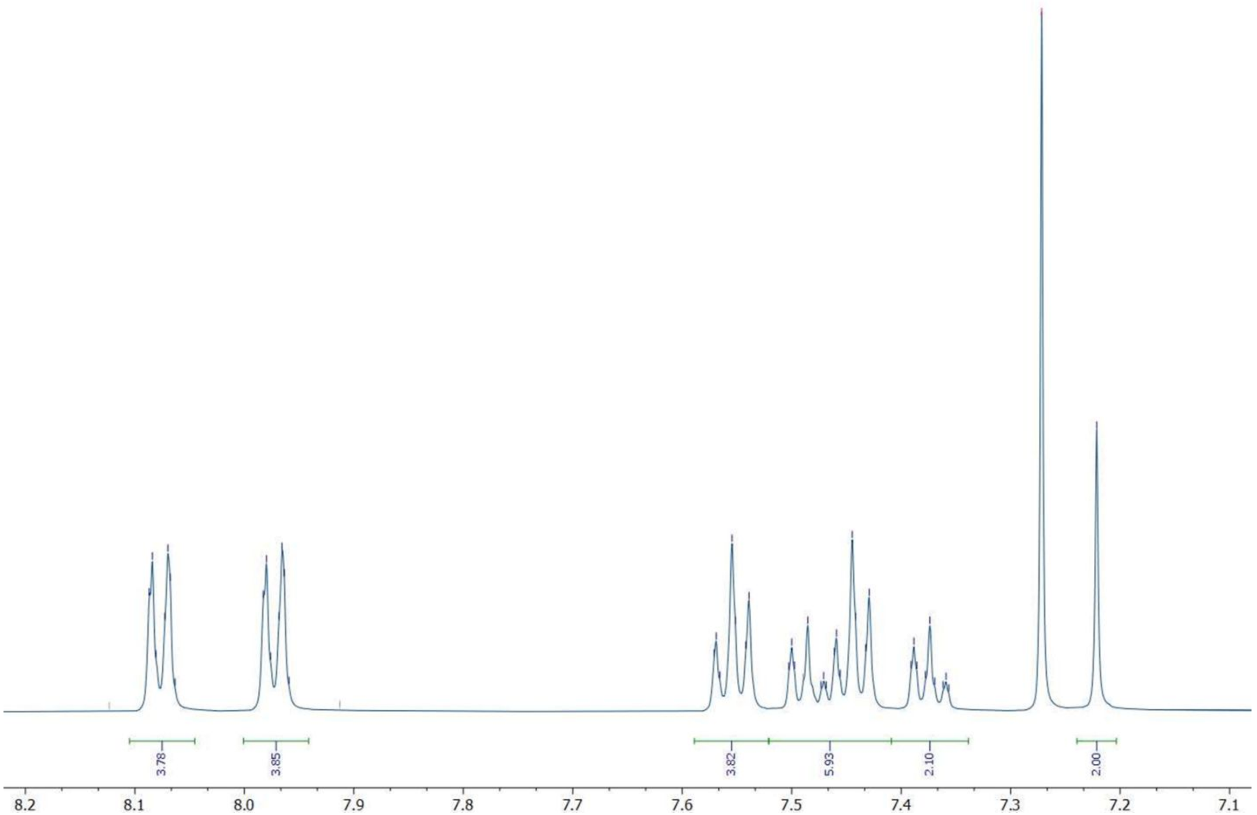

Figure S 5. ^{1}H NMR spectrum of tetraphenyl conjugated azadipyrromethene.

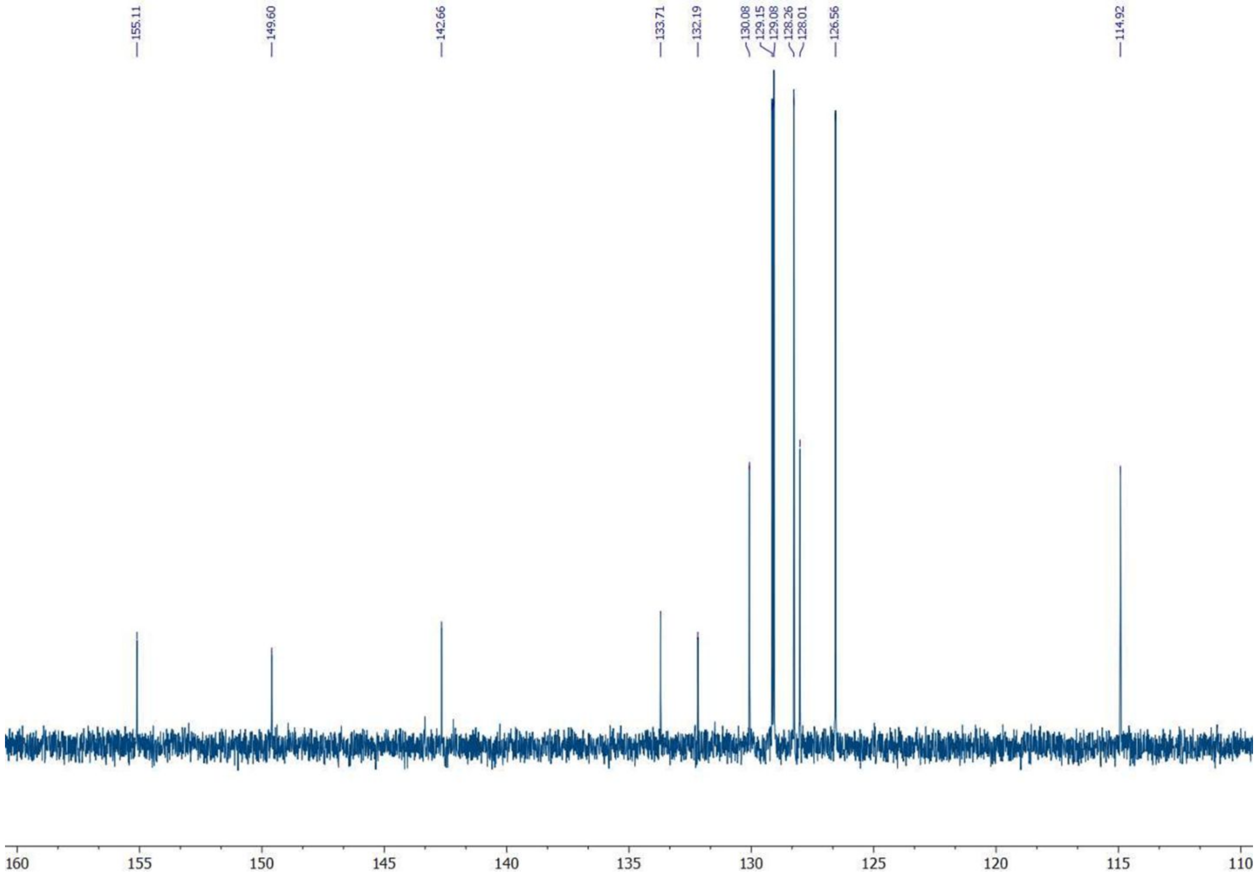

Figure S 6. 13C NMR spectrum of tetraphenyl conjugated aza-dipyrromethene.

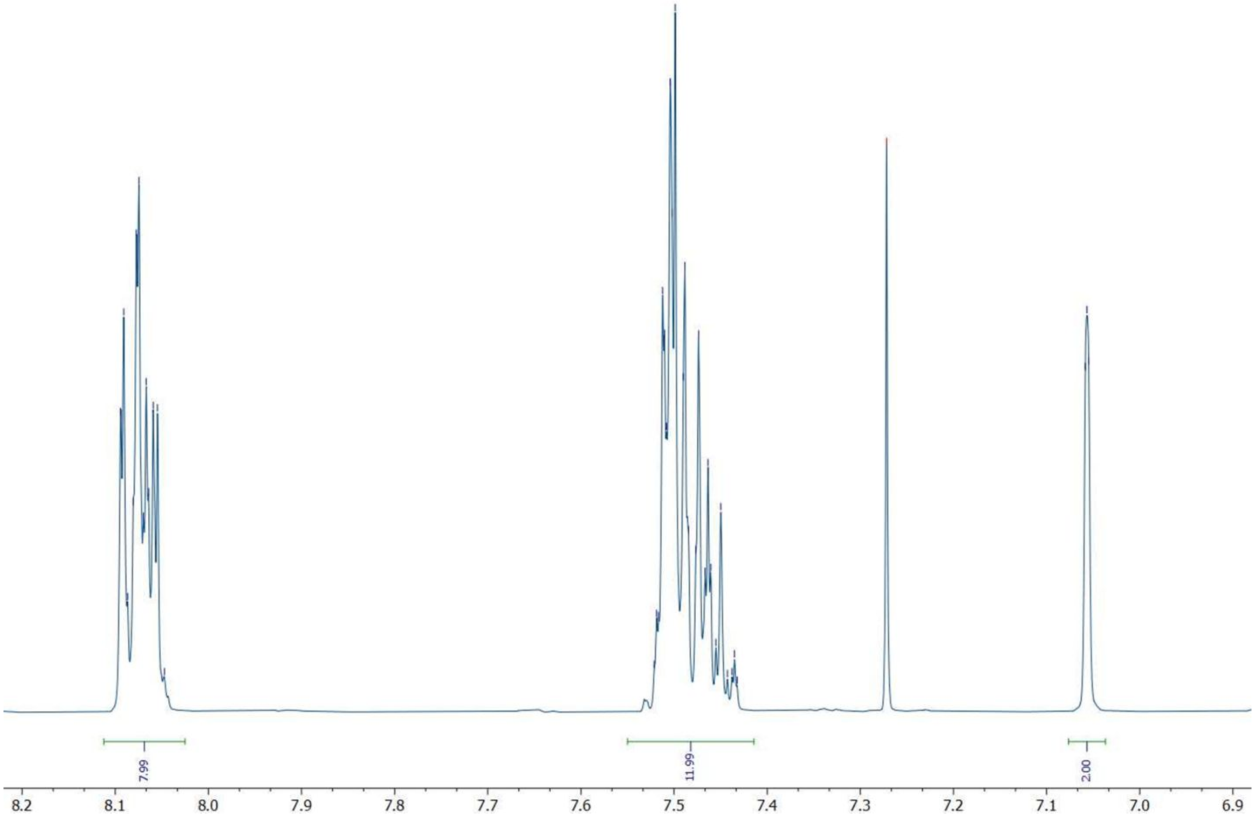

Figure S 7. ^{1}H NMR spectrum of tetraphenyl conjugated aza-BODIPY.

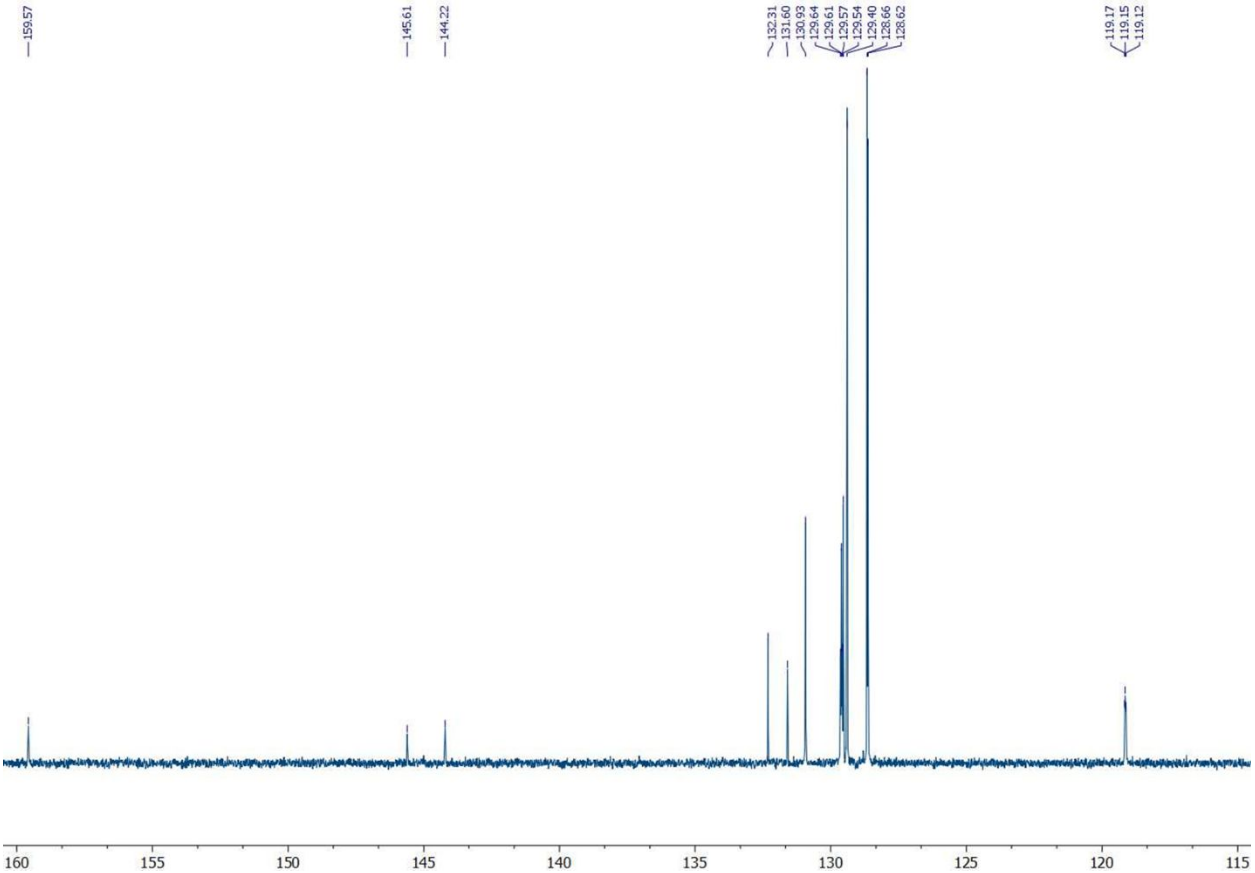

Figure S 8. ^{13}C NMR spectrum of tetraphenyl conjugated aza-BODIPY.

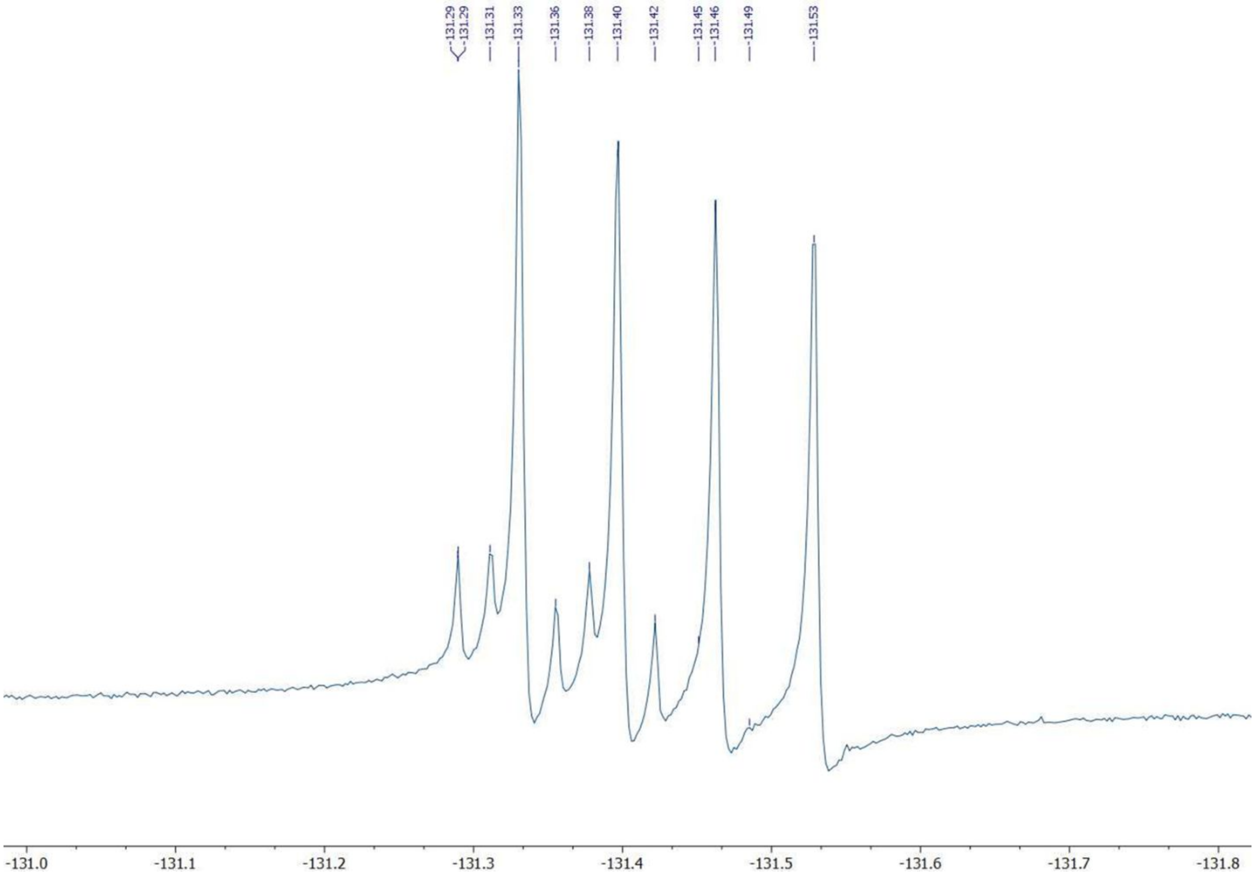

Figure S 9. ^{19}F NMR spectrum of tetraphenyl conjugated aza-BODIPY.

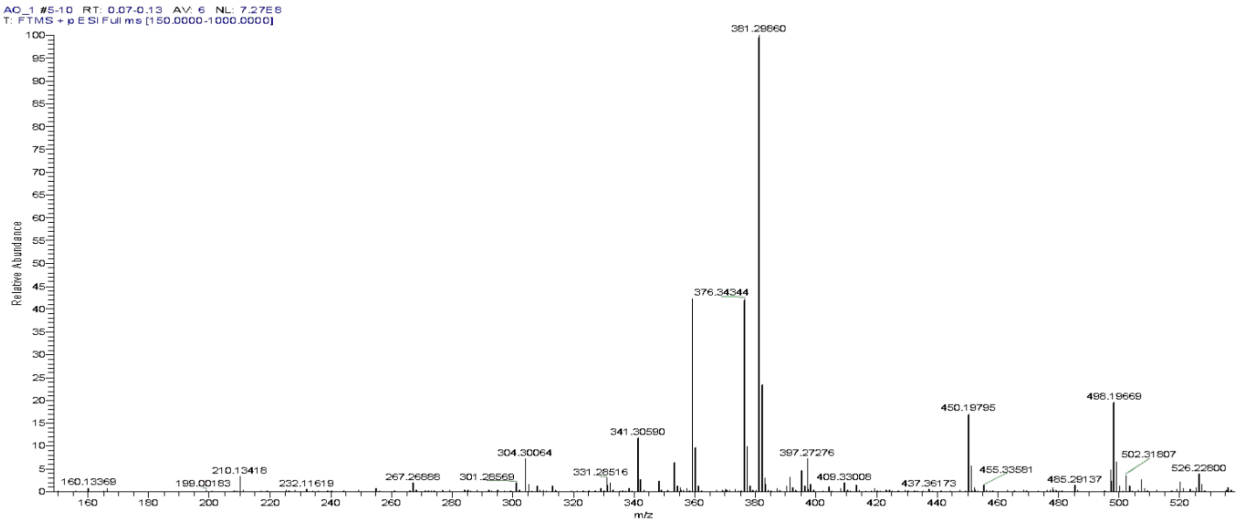

Figure S 10. HR-MS spectra of aza-BODIPY.

2.1 Optical characterization of the sensor

Absorption and Emission Spectra of the sensor: Fluorescence and UV-Vis spectrophotometric measurements were carried out to determine the relevant wavelength and the concentration range for titration analysis (Figure S11 and Figure S12). For that purpose, 1.00 × 10^{-3} M solution aza-BODIPY in acetonitrile was prepared in amber volumetric flask and kept in an ultrasonic bath at room temperature for 30 minutes. This process was repeated before each analysis to avoid precipitation for 5 minutes. Each solution used during analysis was freshly prepared using a stock solution. During the measurements, the excitation wavelength was set as 642 nm meanwhile the slit width was kept constant at 5 nm (excitation) / 10 nm (emission) and the device voltage was adjusted to 600V.

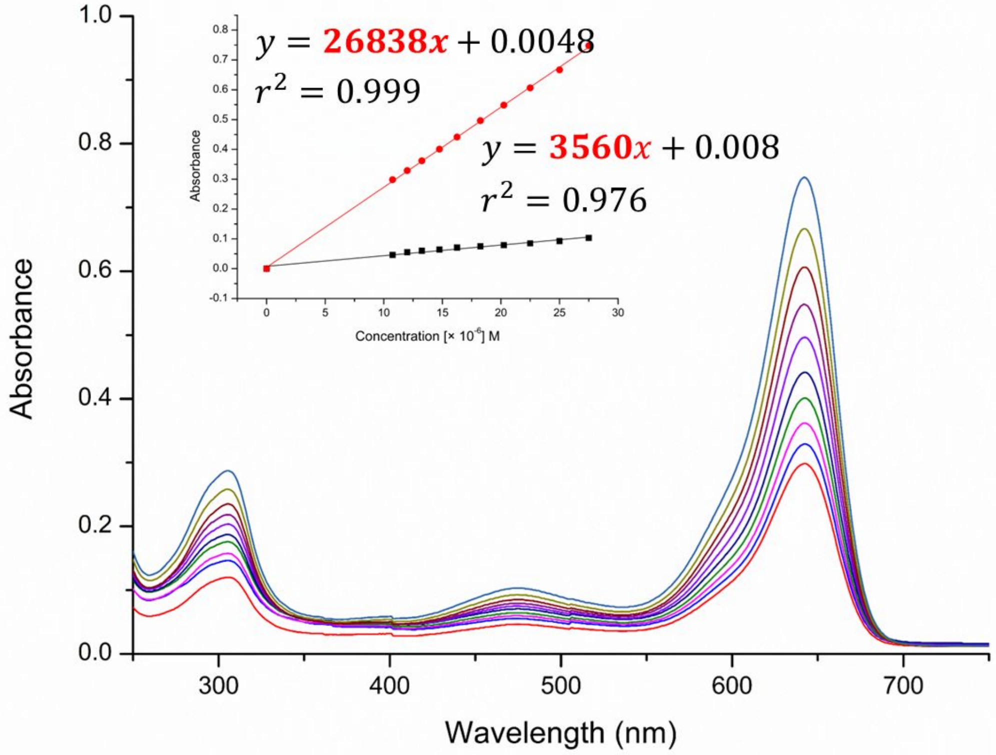

Figure S 11. UV-Vis spectrum of aza-BODIPY in acetonitrile (Concentration between 10.00-30.00 μM).

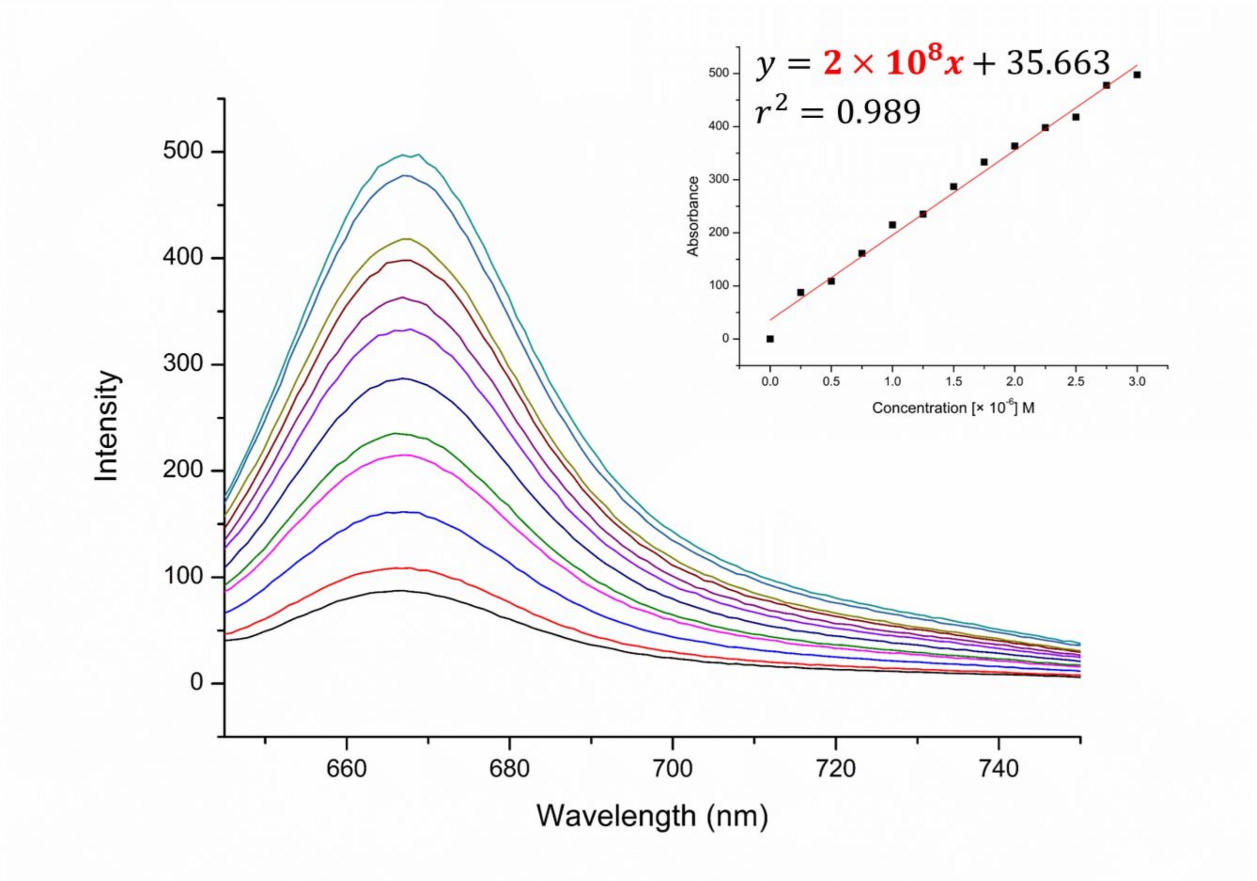

Figure S 12. Fluorescence spectrum of aza-BODIPY in acetonitrile ($\lambda_{excitation}$ = 642 nm, concentration between Concentration between 0.25-3.00 μM).

Fluorescence Lifetime Decay: The lifetime decay of aza-BODIPY was measured to explain the quenching mechanism of the interaction with the explosive. For this purpose, lifetime decay curves of both neat and TNP interacted aza-BODIPY solutions were given in Figure S14.

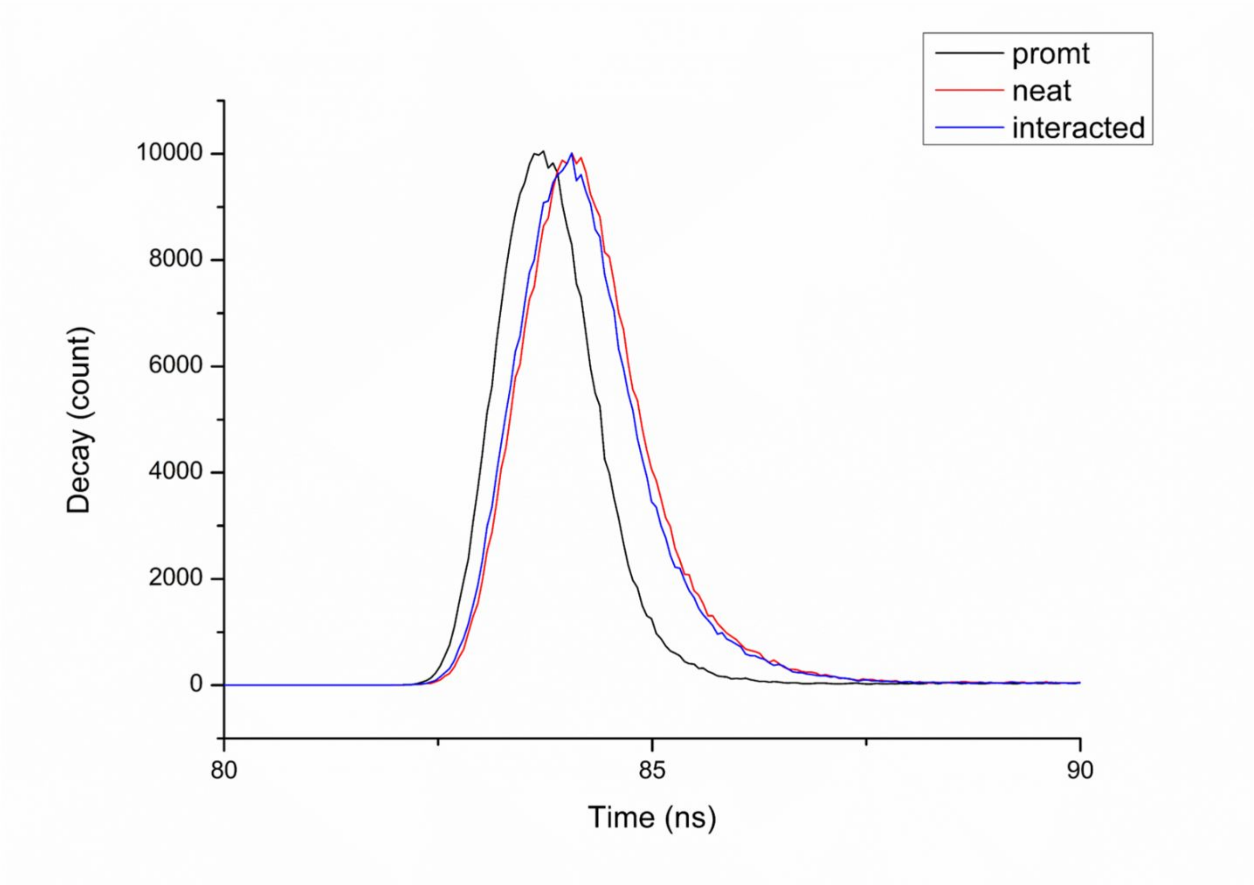

Figure S 13. Fluorescence lifetime of aza-BODIPY.

Limit of Detection: The LOD value, represents the lowest concentration of analyte that can be detected by the corresponding chemical sensor. The graphs of fluorescence intensity values against increasing explosive concentration are shown in Figure S14. The slope values of these graphs are used in the limit of detection (LOD) calculation with the following equation: $LOD = \frac{3 \times \sigma_{10}}{m}$, where σ_{10} is the standard deviation of the fluorescence intensity of the bare aza-BODIPY and m is the slope of the calibration curve drawn according to the fluorescence quenching at the maximum emission wavelength of the analyte. LOD values are listed in Table S1.

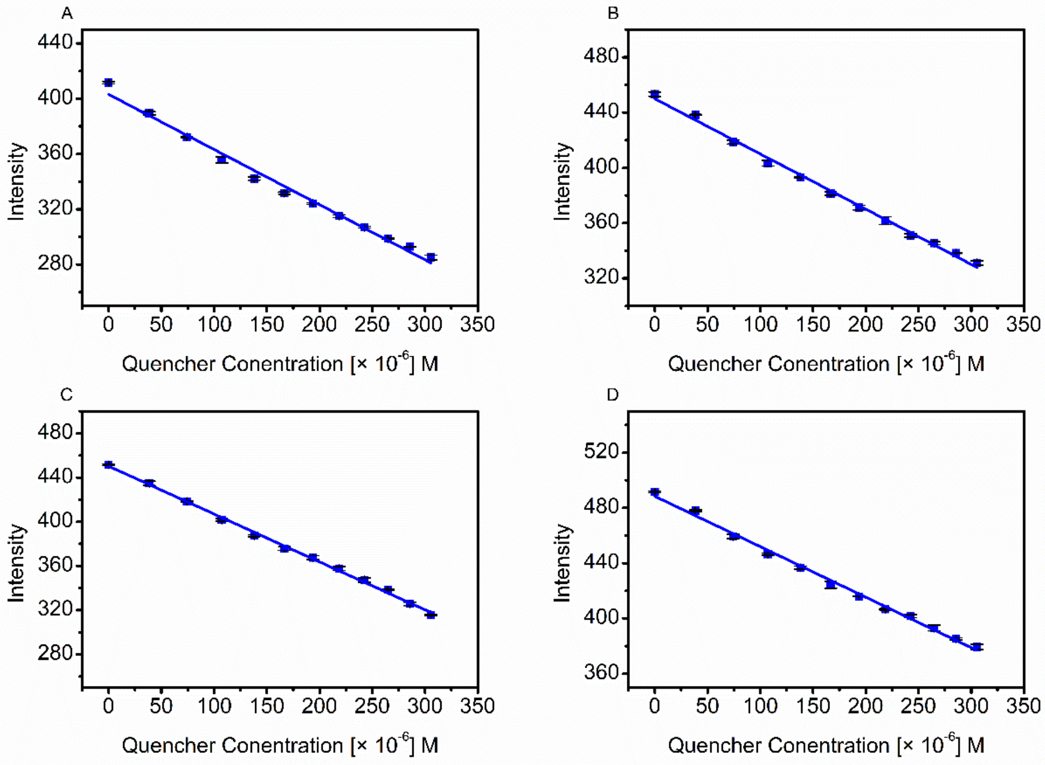

Figure S 14. Calibration curves of titration of aza-BODIPY in acetonitrile with a solution of TNP in water (A), TNP in ethanol (B), TNT in ethanol (C) and DNT in ethanol (D).

Table S 1. Limit of Detection Values.				
	TNP (water)	TNP (EtOH)	TNT (EtOH)	DNT (EtOH)
slope	-399025	-399244	-433052	-365208
σ_{10}	0.3089			
LOD	2.32×10^{-6}	2.32×10^{-6}	2.14×10^{-6}	2.54×10^{-6}

2.2 Computational Details

The theoretical absorption spectra of aza-BODIPY with or without NACs along with the graph corresponding to the spectral overlap between the theoretical fluorescence spectrum of aza-BODIPY with the absorption spectra of NACs were calculated by using Time-dependent DFT (TD-DFT) calculations and shown in Figure S15 and S16. The frontier orbital energies for aza-BODIPY and NACs were calculated at B3LYP level of theory in polarized double zeta basis. The results were summarized in Table S2.

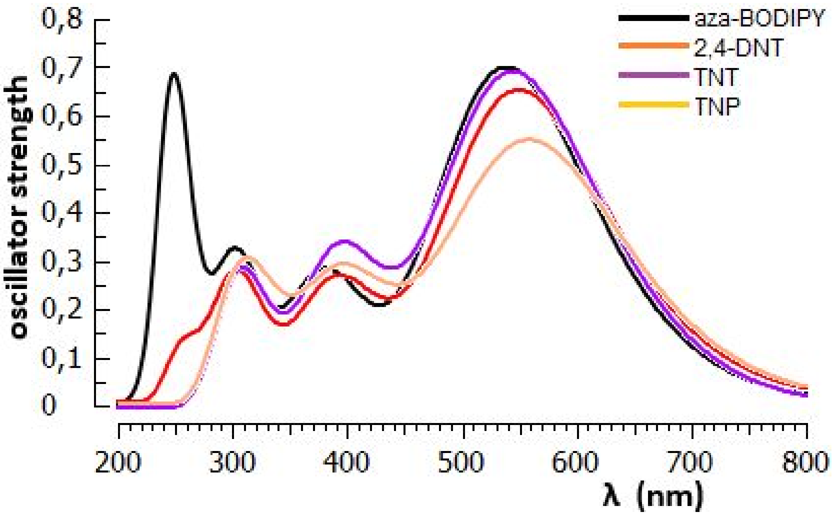

Figure S 15. Theoretical absorption spectra of aza-BODIPY with or without NACs.

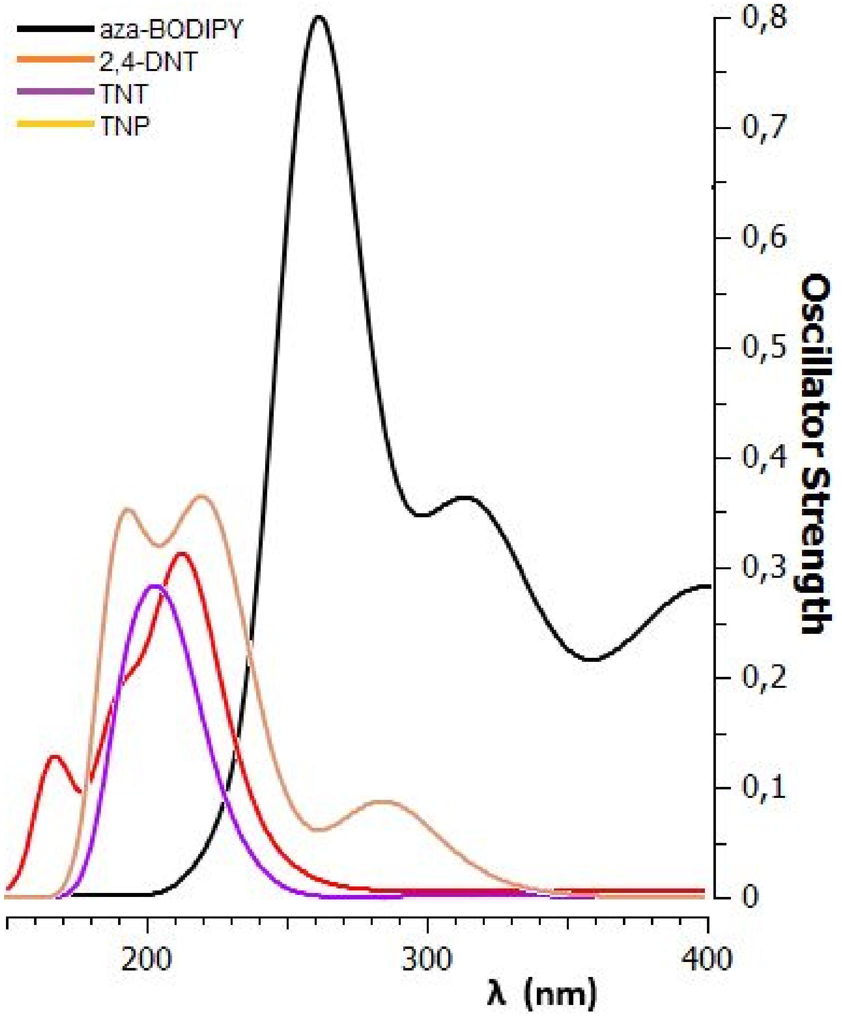

Figure S 16. Spectral overlap between the theoretical fluorescence spectrum of aza-BODIPY with the absorption spectra of NACs.

Table S 2. The frontier orbital energies for aza-BODIPY and NACs calculated at B3LYP level of theory in polarized double zeta basis (in eV).

	E_{HOMO}	E_{LUMO}	Energy gap
aza-BODIPY	-5.35	-3.16	2.19
2,4-DNT	-8.11	-2.98	5.13
TNT	-8.46	-3.49	4.97
TNP	-8.23	-3.92	4.31
